# Do not make me roll initiative: Assessing the Big Five characteristics of Dungeons & Dragons players in comparison to non-players

**DOI:** 10.3389/fpsyg.2022.1010800

**Published:** 2022-10-19

**Authors:** Timo Lorenz, Leonie Hagitte, Melvin Brandt

**Affiliations:** Department of Psychology, MSB Medical School Berlin, Berlin, Germany

**Keywords:** personality, Big Five, nerd, geek, geek-culture

## Abstract

The so-called geek-culture becomes increasingly more mainstream, and its social and economic impact is growing. In contrast, there is very little quantitative psychological research on this subculture and the people immersed in it. The aim of this study was to investigate whether there are differences in the Big Five personality factors between Dungeons & Dragons players and non-players. Within a sample of 801 individuals – 399 Dungeons & Dragon players and 402 non-players - the results indicated that Dungeons & Dragons players show statistically significant higher scores in extraversion as well as in openness to new experiences. Furthermore, we found a statistically significant correlation between Dungeons & Dragons players’ scores in extraversion and their character’s charisma. The results are in line with more recent findings from Big Five research in the geek-culture and contradict older findings regarding a low extraversion and high neuroticism of Dungeons & Dragon players.

## Introduction

Although tabletop games such as Dungeons & Dragons (D&D) are seen as being an unusual hobby, it is amongst other formerly marginalized or obscure considered hobbies (e.g., comic books, cosplay etc.) becoming progressively mainstream (McCain et al., 2015). Considered as being part of the *geek-culture*, these hobbies belong to a highly influential, though rather poorly psychologically researched subculture (McCain et al., 2015; Finister et al., 2021). In the beginning, it was incorrectly associated with teenage delinquency and immorality by the mainstream media (Baker et al., 2022). Moreover, there is a stigma associated with D&D players which persists until today (Sidhu and Carter, 2020; Polkinghorne et al., 2021) based on its purported connections to satanism and other controversies (Baker et al., 2022).

### Dungeons & Dragons

*Role-playing games* (RPG) are cooperative games involving the players and the game master. The game master’s job is the creation of the setting and to provide a basic framework for a story. This person also embodies all fictional characters not embodied by the players and interacts with the players. The players, on the other hand, usually embody one fictional character each. This character has been created by the player, in that certain personality traits as well as behaviors were attributed to them. Additionally, each fictional character has different abilities that help them accomplish certain tasks, the game master sets, within the fictional world. These abilities are expressed through various *stats* determining how, e.g., strong, dextrous, intelligent, or charismatic a character is. During the game, players embody their characters and *as them* they interact with the environment and the game world.

Because of the different abilities available to the character, there are many ways to play the game. A player who is very focused on the role-playing aspect of D&D might give his character more abilities that are social in nature and help the character to intimidate or deceive others and avoid combat if possible. For such social characters, the Charisma attribute plays a particularly important role. Again, a player who for example wants to play a more fight focussed character makes sure that the character is skilled in using weapons and can last as long as possible in combat. Of course, there are several gradations between these two preferences. How good a character is at one thing or the other is determined, at least in part, by the “*Class*” (e.g., Wizard, Druid, Fighter, or Rogue) and “*Species*” (e.g., Human, Elf, or Dwarf) of the character.

### D&D and nerd/geek-culture

D&D consistently requires the players to do math in their head (e.g., determining the hit points or adding stat-boni). This and its role-playing component, resulted in its label as a *nerd* or *geek* game and was considered more of a niche hobby for a long time. This often attributed certain personality factors to the players. The term nerd alone already indicates an initial personality attribution. This is because nerds are often stigmatized as “socially incompetent.” Through this statement, one could infer lower scores on the extraversion personality factor, should it be true. This would fit the image and stereotype of the introverted nerd who is reserved and only interested in his hobby.

Especially in the early days of D&D, players were often associated with Satanic practices (Martin and Fine, 1991). Religious institutions criticized that rituals within the game were too realistically modeled after Satanism (Schnoebelen, 1989). Schnoebelen (1989) also tried to show in his article that D&D “brainwashes” the players and tries to relate increased numbers of suicides to D&D. Schnoebelen does not use any sources or evidence for his statements–nonetheless, we include this article because it reflects the general image and prejudices religious groups had of D&D at that time, especially in the United States. Both geek and nerd were pejorative terms in the times the mentioned articles above were published. Despite this, geeks and nerds in the 80’s began to use those names in some form of self-labeling, expressing pride in their membership in those subcultures (Tocci, 2009; Woo, 2012; McCain et al., 2015).

Acknowledging the self-labeling and in contrast to the derogatory use of those terms we will continue to use the terms geek and nerd to speak of participants in nerd/geek-culture. In that regard the acknowledgment of the self-labeling is meant to highlight the understanding that when speaking of subcultures, as well as when speaking of marginalized people, the power should always be ensured to lie with the people of those groups, respectively.

### D&D in other settings

Moreover, potential other fields to use D&D and other RPGs are getting more attention nowadays. One example is the use of D&D in therapeutic settings. Abbott et al. (2022) discussed the use of D&D in a therapeutic intervention. They treated individuals experiencing social anxiety, and found that participants reported feeling more confident in social situations, especially when making mistakes or regarding boundaries and unexpected events. Furthermore, the authors demonstrated that these skills were transferred to the real world. Another example for exploiting potential beneficial effects of D&D was discussed by Wright et al. (2020). They examined the influence of imaginative role-playing on moral development and suggest that “imaginative role-play gaming structures can function as an engaging, interactive “moral training ground,” a medium that promotes moral development” (p.1). Additionally, in recent years, during the COVID-19 pandemic, new aspects of role-playing have become relevant. Baker et al. (2022) reviewed the potential benefits on mental health of games such as D&D especially regarding the raised demands during the COVID-19 pandemic and suggested the use of RPGs “as an intervention-based approach for the improvement of mental health. Beyond the clinical use, Baker et al. (2022) state that RPGs could also yield beneficial effects when used as a game-based learning method. They specifically highlight RPGs potential to facilitate the learning/teaching of probability theory, basic statistics, and exploring ethical dilemmas. Participants in RPG-based interventions report an increase in self-efficacy and Bowman and Lieberoth (2018) argued that this is all the more an example why RPGs could be a tool in developing impulse control and social competences.

Taking into account the criticism of Polkinghorne et al. (2021), Baker et al. (2022) suggested that “an inclusive environment should be engendered to support the mental health and/or learning of players” (p.7). Conclusively, the currency of using parts of geek-culture in therapeutic settings becomes apparent.

### Personality factors of D&D Players

Personality and traits of character are an essential part of RPGs. And therefore have been in the interest of psychological studies for some time (Bowman and Lieberoth, 2018). Personality can be measured using a variety of methods and there are several classifications that are applied depending on the test used. However, by far the most widely used model is the Five Factor Model (Allport and Odbert, 1936), also called the *Big Five*. These factors have been substantiated in many studies and are now common practice in personality research (John et al., 2008).

Even though a lot of negative attributions and assumptions were made towards players, especially in the early days of D&D, there is very little scientific literature regarding them. Only a few studies have examined the personality factors of D&D players. However, many of the studies have also failed to reach a consistent conclusion, thus creating a varying picture of the personality of RPG players and D&D players more specifically (Bowman and Lieberoth, 2018). Studies comparing extraversion between D&D-players and non-D&D-players, report contradicting results. Douse and McManus (1993) reported that D&D players tend to have lower scores in extraversion. Carter and Lester (1998), on the other hand, found no differences in extraversion between the two groups, while McCain et al. (2015) even showed that D&D players have potentially higher extraversion than non-players. Even when abstracting the topic and expanding the very narrow field of study of D&D to the broader field of the geek-culture or the field of science, technology, engineering and mathematics, few studies address the interindividual differences in personality or compare them to other groups.

To our knowledge, McCain et al. (2015) are the only scholars examining various facets of geek-culture and associated personality factors. Among other things, they found that participating in this culture promotes and fulfills needs of belongingness for creative expression. The relevance of personality and identity in the context of RPGs and therefore in D&D as well, becomes furthermore evident when looking at the concept of immersion in RPGs. The concept of immersion has been troubled in the past and is not viewed uniformly in the community (Bowman and Lieberoth, 2018). Although there is some dissent on how the concept of immersion should be framed or whether or not this concept even should be abolished, for playing D&D, fantasy and immersion are important factors (Bowman and Lieberoth, 2018). Immersion is a state of mind created when the players entirely stay in character and many players report those experiences of immersion by using phrases such as “losing myself in the game” or “the character took over” (Bowman and Lieberoth, 2018). Introverted individuals may exhibit negative feelings, speaking or roleplaying in front of others, which could intensify when acting as a character a person does not feel connected to.

### Hypotheses

McCain et al. (2015) found that individuals who are active in the geek-culture have statistically significantly higher scores in openness to new experiences. This is explained by the fact that geek-culture is far from mainstream. They also found statistically significantly higher scores in extraversion and neuroticism among geeks. There are no differences between agreeableness and conscientiousness. From this, the following hypotheses can be generated:

*H1.1*: *Dungeons* & *Dragons* players report a higher extraversion than non-players.

*H1.2*: *Dungeons* & *Dragons* players report a higher openness to new experiences than non-players.

*H1.3*: *Dungeons* & *Dragons* players report a higher neuroticism than non-players.

*H1.4*: There are no statistically significant differences between the two groups in either conscientiousness or agreeableness.

*H2*: There is a positive association between extraversion in *Dungeons* & *Dragons* players and the charisma attribute of their characters.

## Materials and methods

We report every outcome variable we assessed in this section. No other dependent variables have been included or excluded in the process of this study.

### Sample description and acquisition

A sample of 801 participants was available for the study of the first hypothesis block (H1.1–H1.4). The age of the participants ranged from 15 to 69 years with an average age of 28 years (*SD* = 9.00). The gender distribution within the sample was balanced with 46.1% of participants being men, 46.6% of participants being women, and 7.3% participants reporting a non-binary gender. Participants’ educational attainment varied: 34.2% (*n* = 274) reported having a bachelor’s degree, 32.6% (*n* = 261) had obtained a high school or similar degree, and 15.1% (*n* = 121) had a master’s degree. A large proportion of the participants were single (43.6%), in a relationship (26.3%), or married (19%). The occupational field in which the surveyed subjects work was inquired using the English translation of the KldB2010 (German Classification of Occupations, Bundesagentur für Arbeit, 2011). The distribution of the professions practiced is balanced between the different possible fields of work, e.g., from IT, finance, social or educational sector to humanities.

In order to generate as large and representative a sample as possible, the subjects were acquired through various channels in a snowball sample system. At the end of the survey, participants were asked to forward the survey link to people they knew. Participation was voluntary; hence, no incentives were supplied. Most of the subjects were recruited through various social networks (e.g., Facebook, LinkedIn). For the recruitment of D&D players, specific groups were contacted on Facebook or a study note with a link was posted in player forums. The same sample was used for the last two hypotheses, only all non-D&D players were filtered out for this purpose. The sample of D&D players consists of 399 subjects. Of the 399 subjects, 233 (58.4%) are men and 136 (34.1%) are women; 24 participants (6%) identified as non-binary. Most of the subjects have either a bachelor’s degree (36.3%) or a high school diploma (27.1%). 153 (38.3%) of the subjects are single, 105 (26.3%) are in a relationship, and 95 (23.8%) are in a marriage. Again, the age range of the sample is 15–64 years old with a mean age of 30 years (*SD* = 9.4 years). The non-players had a mean age of 25.8 years (*SD* = 8.1). The control group consists of 403 subjects, of whom 59.1% are women and 33.8% are men; 26 participants (6.5%) identified as non-binary gender. Most of the participants have a high school diploma (38.2%) or a bachelor’s degree (32.0%). The most participants are single (48.6%) or in a relationship (26.3%). The data was collected in English and with the online tool Unipark. Due to forced choice in the standardized questionnaires, there was no missing data.

### Personality

We assessed personality factors using the English version of the BFI-44 (John et al., 1991; Lang et al., 2001). The BFI-44 is a questionnaire with 44 items, which assess the degree of agreement to various statements using a five-point rating scale. Openness is assessed using 10 items with a Cronbach’s α of 0.78 and McDonald’s ω of 0.94. Conscientiousness is assessed using nine items with an α of 0.82 and ω of 0.85. Extraversion is assessed using eight items with an α of 0.89 and ω of 0.92. Agreeableness is assessed using nine items with an α of 0.73 and ω of 0.79. Neuroticism is assessed using eight items with an α of 0.86 and ω of.89.

### Additional questions for D&D Players

Individuals were asked whether or not they had played D&D before. D&D players were then asked what charisma score they preferred when playing (“*If you Play Dungeons & Dragons, how high is your Charisma Score (not Modifier) on average*?”) and which class they prefer (“*What Archetype do you prefer to play*?”).

### Data analysis

For hypotheses H1.1–1.4, we used *t*-tests. The data met the requirements, except for being normally distributed. Since *t*-tests are robust to violating the assumption of normal distribution when group size is balanced (Rasch and Guiard, 2004; Pagano, 2010), we continued with our analyses. We computed a total of five *t*-tests for independent samples, with one of the respective Big Five personality factors as dependent variable. The independent variable, i.e., the grouping variable, is the question of whether they have played D&D before. To compensate for alpha error accumulation in the number of tests, we used Bonferroni-Holm correction. To examine H2, we used a sample only consisting of D&D players and calculated Spearman correlations between the preferred charisma score and the reported extraversion in an exploratory approach, we checked mean scores and standard deviations of the Big Five grouped for the preferred classes for anomalies.

## Results

Table 1 gives an overview of the bivariate correlations of the Big Five, age and player status. Our first hypotheses stated that D&D players report higher scores in extraversion. Results show a statistically significant difference, *t*(794) = 4.92, *p* < 0.001, *d* = 0.35 suggesting that D&D players report higher extraversion scores. The H1.2 was to determine if there were differences in openness to new experiences in favor of D&D players. The result again indicates a statistically significant difference between D&D players and non-D&D players (*t*(800) = 5.87, *p* < 0.001, *d* = 0.41) suggesting that D&D players report higher scores in openness to new experiences. For H1.3, the aim was to investigate whether players report higher scores than non-players on the neuroticism factor. The *t*-test showed a statistically significant difference between the groups (*t*(797) = −3.10, *p* = 0.002, *d* = −0.22). Contrary to our predictions, the non-gamer group reports higher neuroticism scores. For H1.4, we expected no statistically significant differences in agreeableness and conscientiousness. The *t*-tests support the hypothesis (agreeableness: *t*(790) = 0.81, *p* = 0.417, *d* = 0.06; conscientiousness: *t*(796) = −0.114, *p* = 0.253, *d* = −0.08). Correlation analysis revealed a statistically significant relationship, *ρ* = 0.18, *p* < 0.001 supporting H3 and indicated that the reported extraversion score is positively associated with the charisma score of the played character.

**Table 1:** Means, standard deviations, and Pearson correlations with confidence intervals of the Big Five domains from D&D players and non-players.

Variable	*M*	*SD*	1	2	3	4	5	6	7
1. Openness	3.80	0.62							
2. Conscientiousness	3.15	0.73	0.02						
			[−0.04, 0.09]						
3. Extraversion	2.91	0.93	0.26^***^	0.13^***^					
			[0.20, 0.33]	[0.06, 0.20]					
4. Agreeableness	3.69	0.60	0.09^**^	0.19^***^	0.19^***^				
			[0.02, 0.16]	[0.12, 0.25]	[0.12, 0.26]				
5. Neuroticism	3.15	0.88	−0.04	−0.20^***^	−0.31^***^	−0.27^***^			
			[−0.11, 0.03]	[−0.26, −0.13]	[−0.37, −0.24]	[−0.33, −0.20]			
6. Age	27.83	9.00	0.09^**^	0.19^***^	0.15^***^	0.12^***^	−0.18^***^		
			[0.02, 0.16]	[0.12, 0.25]	[0.08, 0.22]	[0.06, 0.19]	[−0.25, −0.11]		
7. Status	-	-	−0.20^***^	0.04	−0.17^***^	−0.03	0.11^**^	−0.23^***^	
			[−0.27, −0.14]	[−0.03, 0.11]	[−0.24, −0.10]	[−0.10, 0.04]	[0.04, 0.18]	[−0.29, −0.16]	
8. Education	3.31	1.28	0.15^***^	0.27^***^	0.11^**^	0.11^**^	−0.11^**^	0.39^***^	−0.12^***^
			[0.09, 0.22]	[0.20, 0.33]	[0.04, 0.18]	[0.04, 0.17]	[−0.17, −0.04]	[0.33, 0.45]	[−0.19, −0.05]

*M* and *SD* are used to represent mean and standard deviation, respectively. Values in square brackets indicate the 95% confidence interval for each correlation. The confidence interval is a plausible range of population correlations that could have caused the sample correlation (Cumming, 2014). *indicates *p* < 0.05. **indicates *p* < 0.01. ***indicates *p* < 0.001. Status is a binary variable (1 = player; 2 = non player), referring to whether the participants reported having played D&D or not.

### Exploratory analysis

In an exploratory data analysis, we checked the mean scores and standard deviations of the personality traits clustered into preferred character groups. It seems there is a positive association between extraversion and the class spellcaster (e.g., Wizard, Warlock), as well as a negative one with nature oriented (e.g., druid) classes. These two seem to be the only ones with a slightly higher or lower mean score. For the complete overview see Table 2. To account for those differences, seen in extraversion mean scores, again several *t*-tests with Bonferroni-Holm correction were performed, comparing the extraversion scores of those, who preferred the class spellcaster, as well as those players, who prefered nature themes classes against all other extraversion scores grouped after class-preferences. The tests resulted in two statistically significant results with spellcaster vs. nature themed (*t*(173) = 2.917, *p* = 0.004, *d* = 0.44) and spellcaster vs. multiclass (*t*(168) = 2.071, *p* = 0.040, *d* = 0.32). The rest of the tests were not statistically significant with d ranging from nature themed vs. multiclass (*t*(108) = −0.625, *p* = 0.534, d = −0.12) to nature themed vs. rogue (*t*(139) = −1.689, *p* = 0.094, *d* = −0.29).

**Table 2:** Big Five mean scores and class preferences mean scores.

	Big Five means (*SD*)				
	Openness	Conscientiousness	Extraversion	Agreeableness	Neuroticism
Spellcaster (e.g., Sorcerer, Wizard, Warlock); *n* = 115	3.97 (0.55)	3.08 (0.68)	3.26 (0.85)	3.66 (0.58)	3.17 (0.89)
Divine Classes (e.g., Cleric, Paladin); *n* = 50	3.91 (0.60)	3.11 (0.77)	3.06 (0.79)	3.72 (0.47)	3.16 (0.98)
Nature themed Classes (e.g., Ranger, Druid); *n* = 57	3.99 (0.52)	3.42 (0.71)	2.80 (0.85)	3.80 (0.57)	3.15 (0.99)
Martial Classes (e.g., Barbarian, Fighter); *n* = 45	3.81 (0.51)	3.28 (0.62)	3.10 (0.89)	3.73 (0.57)	2.87 (0.82)
Roguelike Classes (e.g., Rogue, Monk); *n* = 82	3.84 (0.58)	3.22 (0.65)	3.07 (0.85)	3.68 (0.63)	2.95 (0.87)
Multiclass Characters; *n* = 50	4.00 (0.57)	3.16 (0.72)	2.95 (0.90)	3.72 (0.55)	2.91 (0.85)

Furthermore, associations between the character’s charisma score and the player’s personality traits have been tested. Besides the hypothesized correlation with extraversion and the charisma score, two correlations are statistically significant, a negative correlation between conscientiousness, *ρ* = −0.164, *p* < 0.001, and a positive correlation with openness, *ρ* = 0.114, *p* < 0.05. The correlations with agreeableness (*ρ* = 0.016, *p* = 0.751), and neuroticism, (*ρ* = −0.059, *p* = 0.249) were not statistically significant.

## Discussion

The geek-culture is becoming increasingly more mainstream, and its social and economic impact is growing. In contrast, there is very little psychological research on this subculture and the people immersed in it. The present findings are consistent with the results of McCain et al. (2015), but contradict older findings regarding personality characteristics (Douse and McManus, 1993; Carter and Lester, 1998). The results of Douse and McManus (1993), and Carter and Lester (1998) must be viewed critically from today’s perspective. First, the individuals who played at that time are not necessarily the players of today, therefore the results refer to a differently composed group of people. Second, the results of Douse and McManus (1993) are based on a play-by-mail game, compared to the face-to-face or *via* livestream style of playing D&D nowadays, while Carter and Lester (1998) collected a sample of 20 men.

Consistent with the findings of McCain et al. (2015), we found statistically significant differences in reported openness and extraversion scores between D&D players and non-players. Regarding neuroticism, statistically significant differences could be found, however directed contrarily than assumed. Contrary to our prediction, players showed lower scores on the neuroticism dimension than non-players. Thus, in this case the present findings contradict those of McCain et al. (2015).

As predicted, we found no statistically significant differences in reported agreeableness and conscientiousness scores. This suggests that players and non-players do not differ on these personality dimensions, and differences are most likely random in nature.

D&D has been repeatedly revised and renewed since the first versions. With the fifth edition of the rulebook, many simplifications to the rules have been made, making it the most beginner-friendly version of D&D until now. The latest ORR Group industry report (2021) from the site Roll20.net, an online platform for players of various RPGs, reports thatD&D has received a lot of growth in the number of players and therefore, is approaching the mainstream. Taking into account the increasing number of players who are new to the hobby, as well as the beginner-friendly newer version of the game, it could be deduced that the pool of players is becoming more heterogeneous shifting the character of the “average D&D player” over time since the beginning of D&D.

Although D&D has become increasingly popular in recent years (e.g., sales during the COVID-19 pandemic have tripled; Baker et al., 2022), it is still a unique concept that for many may take some time getting used to. Despite the growing community, some people still share the old stereotype of the typical nerd or geek, holding them from trying the game. From the perspective of personality research, another explanation could be their lower degree of openness to new experiences, potentially explaining the statistically significantly higher scores among players. Consequently, playing D&D (or at least trying it once) despite the prejudices might require individuals to exhibit a higher openness to new experiences.

Unexpectedly, we found that players exhibited a lower score in neuroticism, thereby contradicting the existing literature and stereotypical image of geek-culture. The stereotype of a neurotic geek (e.g., Sheldon Cooper, a pop-culture-character representing geeks/nerds as being loners) might be outdated.

Another goal of this study was to explore a possible association between certain preferences during gameplay and character creation with a D&D player’s personality. The present study indicates that players with higher scores in extraversion seem to prefer playing charismatic characters as well, suggesting that players of characters with high charisma scores might prefer a more social and role-playing style of play.

A possible explanation for the association of the player’s extraversion and the character stat charisma might be that, compared to other stats, charisma has a unique aspect. Some stats, such as strength and dexterity are usually informed abilities, i.e., if you want to lift a boulder you roll a dice and add your strength bonus, or you look at your strength, and whether it is high enough to lift the boulder. Charisma is often used very differently in the game. If a player is to attempt a social interaction like diplomacy, it is quite common for a game master to require the player to roleplay this encounter, and make a passing attempt at actually playing out this interaction, before a dice roll is made. Playing a highly charismatic character will likely lead to more of these in-game social interactions where the player is required to roleplay certain situations as their character. This type of play might attract players with a higher degree of extraversion.

Another possible explanation for the association of higher extraversion and D&D-players could be the reward-processing view on extraversion. Extraversion as a construct is often understood as the combination of *agency* (e.g., achievement striving, social dominance etc.) and *affiliation* (e.g., positive affect, enjoying interpersonal bonds etc.). Both components are linked to reward sensitivity (e.g., Depue and Collins, 1999; Wilt and Revelle, 2009; DeYoung, 2010). The appealing feature of the reward-processing approach is its potential to explain the connection of the components comprising trait extraversion. Variation in reward responsiveness could account for the fact that people who tend to be more sociable also often tend to be more outgoing and experience high levels of positive affect (Smillie, 2013). The fact that D&D*-*players seem to be more extraverted could be explained by the fact that a game such as D&D is usually rewarding to the player and therefore plays to the reward responsiveness of extraverted individuals all the more. This could result in the game attracting extraverted people in the first place, but also in keeping them in the community because of the inherently rewarding nature of the game experience. Additionally this reward-responsiveness approach also holds the potential to explain the former discussed observation, that players with higher scores in extraversion tend to play more charismatic characters. Altering the experience of the game for players into one that is even more rewarding for extraverted people.

### Limitations of the study

The use of a nonprobability sample in this study raises concerns about its generalizability. What should be taken into account when evaluating this study’s results, is that they specifically apply to D&D as a game system. Although it is not far-fetched to expect these results to be transferable to other game systems since D&D is considered the “prototype” of RPGs, the findings might not necessarily apply to other systems. A further limitation of the study is the recording of player status as a dichotomous variable. A continuous variable on player experience could generate even more insights into how the geek-culture community may have changed. Additionally, when asking for the preferred archetype, we assumed that the players have, in some way, a preferred play style or a preferred type of character when playing D&D or other game systems. However, the participants were not explicitly asked whether that assumption holds true, or not. Thus, creating a potential for incorrect conclusions based on that assumption. It should also be noted that the quantitative style of this study “naturally” limits the results to numerical values based on samples, in the broader sense. Many aspects of RPGs and character traits especially, incorporate more complex aspects that cannot be fully met by a pure quantitative study alone. Thus this should not be the demand of this study. For a more in-depth consideration on this and related topics in the RPG-context, see Zagal and Deterding (2018). Furthermore, no data regarding place of residence were collected in the demographics. Thus, no distribution and representation of regionality can be related to the population. Even though the FFM has been translated into many languages and has been tested across cultures (McCrae, 2002; Schmitt et al., 2007), critics suggest that it is not the best instrument to assess personality in every culture (Ashton et al., 2004; Funder, 2010). This could result in a potential bias.

## Conclusion and outlook

Research in this field is needed to investigate and possibly refute possible negative stigmas and stereotypes towards geek-culture. Moreover, it would be interesting to investigate whether players who report high scores in extraversion display charismatic behavior in the game or if they are using the dice to determine the result of a social interaction in the game. Furthermore, regarding the potential benefits of D&D on moral development and social skills, it would be interesting, whether extraverted players are more apt to try using charisma as strategy for conflict solving, rather than violent in-game strategies. Such results could be obtained in the form of behavioral observations during RPG game-sessions. Such a setting for future research would also be beneficial to generate insight into the behavioral processes during the game, therefore allowing for a more in-depth evaluation of the trends the present findings suggest. Future research could investigate the effect of the (lack of) fit between the personality of the player and attributes of the played character.

While for many gaming is a hobby, there are already some programs using D&D and similar games as a therapy-augmentation for groups to learn various social skills in a safe environment (e.g., Rubin, 2019; Bean, 2020). With a group of fellow like-minded players, people could find it easier to work on these skills without fearing real-world consequences. Contrary to the previously prevalent stereotype that participants in geek-culture are reclusive, these programs represent the idea that geek-culture could actually improve social skills.

This study contributes to the resolving of the negative stigma of D&D players by showing that players report higher levels of extraversion than non-players. Furthermore, they report to be more open to try new things, and report lower levels of neuroticism and, if they ever were, have long since ceased to be the “reclusive and socially incompetent nerds/geeks” they were stereotyped to be and have become participants in geek-culture.

## Data availability statement

The datasets presented in this study can be found in online repositories. The names of the repository/repositories and accession number(s) can be found at: https://www.psycharchives.org/index.php/en/item/c9207b6a-a609-4ddd-a28c-032279d784d6.

## Ethics statement

Ethical review and approval was not required for the study on human participants in accordance with the local legislation and institutional requirements. The patients/participants provided their written informed consent to participate in this study

## Author contributions

TL and MB contributed to conception and design of the study and wrote the first draft of the manuscript. TL, MB, and LH organized the database. TL and LH performed the statistical analysis. All authors contributed to manuscript revision, read, and approved the submitted version.

## Conflict of interest

The authors declare that the research was conducted in the absence of any commercial or financial relationships that could be construed as a potential conflict of interest.

## Publisher’s note

All claims expressed in this article are solely those of the authors and do not necessarily represent those of their affiliated organizations, or those of the publisher, the editors and the reviewers. Any product that may be evaluated in this article, or claim that may be made by its manufacturer, is not guaranteed or endorsed by the publisher.
